# A Visualization Method for Corrosion Damage on Aluminum Plates Using an Nd:YAG Pulsed Laser Scanning System

**DOI:** 10.3390/s16122150

**Published:** 2016-12-16

**Authors:** Inbok Lee, Aoqi Zhang, Changgil Lee, Seunghee Park

**Affiliations:** 1 Environment Technology and Safety Technology Convergence, Inha University, Incheon 405-751, Korea; yangjs0605@hanmail.net; 2 Department of Civil & Environmental System Engineering, Sungkyunkwan University, 2066, Seobu-ro, Jangan-gu, Suwon-si, Gyeonggi-do 440-746, Korea; zaq623@skku.edu (A.Z.); tolck81@skku.edu (C.L.); 3 School of Civil & Architectural Engineering, Sungkyunkwan University, 2066, Seobu-ro, Jangan-gu, Suwon-si, Gyeonggi-do 440-746, Korea

**Keywords:** non-destructive testing, corrosion, pulsed laser scanning, ultrasonic waves, plate structure

## Abstract

This paper proposes a non-contact nondestructive evaluation (NDE) technique that uses laser-induced ultrasonic waves to visualize corrosion damage in aluminum alloy plate structures. The non-contact, pulsed-laser ultrasonic measurement system generates ultrasonic waves using a galvanometer-based Q-switched Nd:YAG laser and measures the ultrasonic waves using a piezoelectric (PZT) sensor. During scanning, a wavefield can be acquired by changing the excitation location of the laser point and measuring waves using the PZT sensor. The corrosion damage can be detected in the wavefield snapshots using the scattering characteristics of the waves that encounter corrosion. The structural damage is visualized by calculating the logarithmic values of the root mean square (RMS), with a weighting parameter to compensate for the attenuation caused by geometrical spreading and dispersion of the waves. An intact specimen is used to conduct a comparison with corrosion at different depths and sizes in other specimens. Both sides of the plate are scanned with the same scanning area to observe the effect of the location where corrosion has formed. The results show that the damage can be successfully visualized for almost all cases using the RMS-based functions, whether it formed on the front or back side. Also, the system is confirmed to have distinguished corroded areas at different depths.

## 1. Introduction

Nondestructive evaluation (NDE) techniques have been successfully applied for the past several decades in various fields, including civil, mechanical, and aerospace engineering [1]. However, the structural integrity of some structures with restricted accessibility and high-precision geometry, such as nuclear power plants, is difficult to assess. Specifically, the most frequent issue encountered in structural health monitoring (SHM) of such structures is the development of corrosion. Corrosion jeopardizes structural safety when it grows to a critical size, and it can occur not only on the outer surfaces of structures, but also inside metal plate structures. Since damage to the back side of a metal plate is difficult to detect, damage detection methods for the back sides of structures are strongly required. This issue can be overcome using economical non-contact sensors, such as the classical SHM/NDE method that depends mostly on actuators and piezoelectric (PZT) sensors [2,3]. Such method can be used to measure wave signals to detect damage using installed PZT sensors [4,5,6,7,8], which are inexpensive.

Effective corrosion detection methods in SHM should be intuitive, and the results should be easily understandable. To this end, some researchers have developed acoustic and ultrasonic wave technologies, including laser vibrometry, laser interferometry, and pulsed lasers. In a previous study [9], a Lamb wave was generated in a duralumin plate immersed in water. The laser Doppler vibrometer (LDV) that was used in that study produced a laser beam perpendicular to the scanned surface. Changes in scan points can be achieved by moving the laser head. In 2003, Kehlenbach et al. [10] published numerical results showing that Lamb waves can be propagated in an aluminum plate. They confirmed those results using a LDV and successfully visualized the propagating waves in a damaged area. Their results showed that the damage detection efficacy depends on the size of the damaged area. Other experimental results [11,12,13] have used Lamb waves to detect damage to aluminum plates by scanning the surface around the damaged side using a LDV. Due to their low noise and narrow line widths (on the order of a few millihertz), those studies used a single-mode HeNe laser as the preferred light source for the LDV. Damage was successfully detected by finding areas with the maximum signal values.

A PZT sensor can also be used in LDV scanning, and the biggest advantage of using an LDV-PZT system is that any arbitrary waveform can be applied at the excitation location of the laser point with the highest energy level. However, even though some aspects of LDV systems have improved, such as automatic focusing and laser spot visibility when measuring very small objects [14], there are still some disadvantages. For example, if the LDV scans the target structure too quickly, speckle noise, an optical phenomenon, could be unavoidable [15]. Furthermore, in LDV-PZT method, it is very important for the LDV sensitivity to depend on the target surface condition and the incident angle of the laser beam. Therefore, it is advantageous for the laser beam to be perpendicular to a surface of the structure because most of the signal produced by the incident laser can be reflected straight back to the PZT sensor. However, when the laser beam scans a large area, the incident angle increases, and the intensity of the produced signal decreases, which will affect the quality of the signal. The signal-to-noise ratio (SNR) of the photodetector output is another limiting factor of LDV [16], due to the low sensitivity of LDV, so multiple ultrasonic signals must be obtained at a single point, and these need to be averaged to improve the SNR [17].

Other researchers have proposed using LDV with an air-coupled transducer (ACT) for NDE when scanning large surfaces. This method has effectively detected various types of damage [18]. However, this transducer has a relatively low sensitivity due to the large mismatch in the acoustic impedance between air and the solid material as well as the high ultrasonic attenuation in air [12]. In addition, two separate lasers have been used to implement a full non-contact damage detection technique, such as using a fixed Nd:YAG pulsed laser as an excitation laser at the same time as an LDV to scan the target area [17,19]. However, this method requires averaging the signal to improve the SNR due to an obviously reduced ultrasonic wave signal. In addition, it is also necessary to control the laser intensity, including the pulse energy, laser diameter, and wavelength, to prevent burning the target structure. Researchers should also pay attention to the input waveform of the laser excitation, which is usually limited to a pulse laser [20,21]. To address those issues, an Nd:YAG pulse laser system can be used to generate ultrasonic waves that can be received by a PZT sensor. In contrast with other technologies, this laser pulse system has many advantages including fast wave generation with a low pulse energy, high spatial resolution, and good detection in complex structures, especially to scan a large area because it is affected little by the incident angle of the laser beam and the irregular surface condition [22]. Often, the allowable incident angle for the excitation laser is of up to ±70 degrees and ±20 degrees for the sensing laser [23].

Many techniques have been developed using LDV, Nd:YAG pulsed laser, ACT, and PZT systems for damage detection by implementing filtering methods and wavenumber-based methods. Ultrasonic wave imaging has been achieved using methods based on wave propagations, and the results have shown good detection abilities for various kinds of damage [24,25,26,27]. In recent years, a new method was developed using a wavefield generated on the structure surface with a scanning laser or an externally scanned transducer, and many studies have verified the feasibility of this method for damage detection [28,29,30,31,32,33]. Several signal processing methods can be used to detect damage through damage-induced reflected and scattering waves. In this study, the non-contact NDE technique is applied by generating ultrasonic waves using a laser to visualize corrosion damage on aluminum plates. The ultrasonic waves are generated with an Nd:YAG pulse laser, and a galvanometer-based laser scanner is used to scan a specific area on the target structure. At the same time, an ultrasonic wave propagation image (UWPI) is obtained by measuring the wave responses with a PZT sensor attached to the central position on the back side of the target structure. The advantages of using such method are the high spatial resolution, ability to scan large complex structures, and ease of recognition.

Structural damage can be visualized by calculating the root mean square (RMS) values of the measured wavefield-time signals at every laser excitation point [34,35]. In order to improve the quality of the RMS snapshots, the authors propose to use the logarithmic values of the RMS function. Not only that, a weighting parameter can obviously improve the color of the RMS snapshots and compensate for the attenuation caused by the geometrical spreading and wave dispersion. The RMS value has the following advantages: (1) it is possible to pinpoint the damage locations; (2) in practical applications, the RMS snapshots can be produced in a relatively short time; (3) this method can effectively classify the degree of damage.

Few studies have used this technology to detect corrosion on internal structures or on the backs of structures, so the authors scan both sides of the plate using the same scanning area. In addition, few research has previously considered the effects of both the corrosion depth and corrosion size. The corrosion conditions for different depths and sizes are also discussed to verify the abilities of this method, including fast scanning of a large area, sensitive locating for corrosion detection, and effective classification of the damage degree. The major focus is on the experimental analysis.

## 2. Ultrasonic Wave Propagation Imaging System

**Figure 1:**
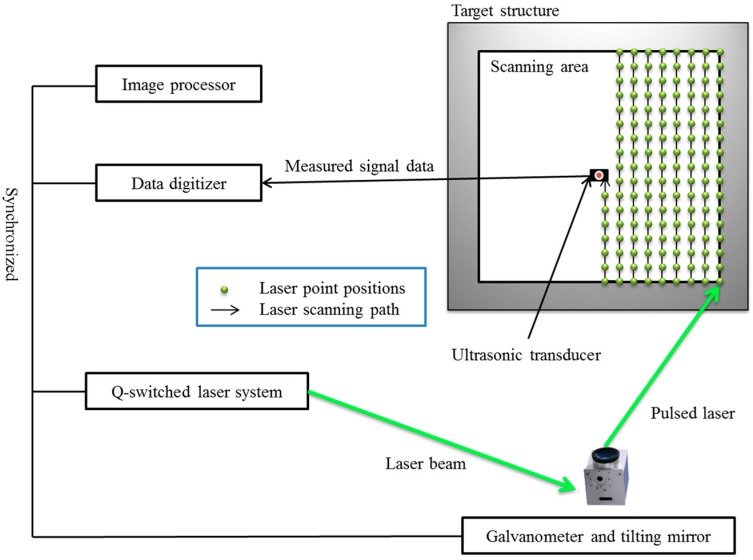
Laser-induced ultrasonic wave propagation imaging (UWPI) system.

As shown in Figure 1, the components of the proposed UWPI system include a Q-switched laser system, a galvanometer-based laser mirror scanner, an ultrasound sensor, a high-speed digitizer, and an image processor. This study used a Q-switched diode-pumped high-power solid-state Nd:YAG laser [36] with a wavelength of 532 nm and a maximum pulse repetition rate of 20 Hz. The energy of each pulse is 0.658 mJ, the diameter of the laser beam is 0.45 mm, and the energy density is 4.15 mJ/mm^2^. The laser mirror scanner can adjust the pulse laser scans for a specified location. The objective of this design is to operate two galvanometers at a wavelength of 532 nm. To ensure that the laser beam can efficiently scan the 2-D area of the target structure, the operating angles of the galvanometer are orthogonal to each other. The f-theta lens is installed at the end of the laser scanner system to reflect the laser beam, which focuses on the target detection area. As shown in Figure 1, the laser beams scan the target area vertically in the horizontal direction, and the laser scanning path can be designed by the image processor. Furthermore, the spacing of each laser impingement point can also be adjusted by the laser control system. Since the laser intensity is adjusted over an appropriate range (1%), the pulsed laser cannot burn or damage the specimen. At the same time, a reduction in the laser intensity can prolong the life of the device.

**Figure 2:**
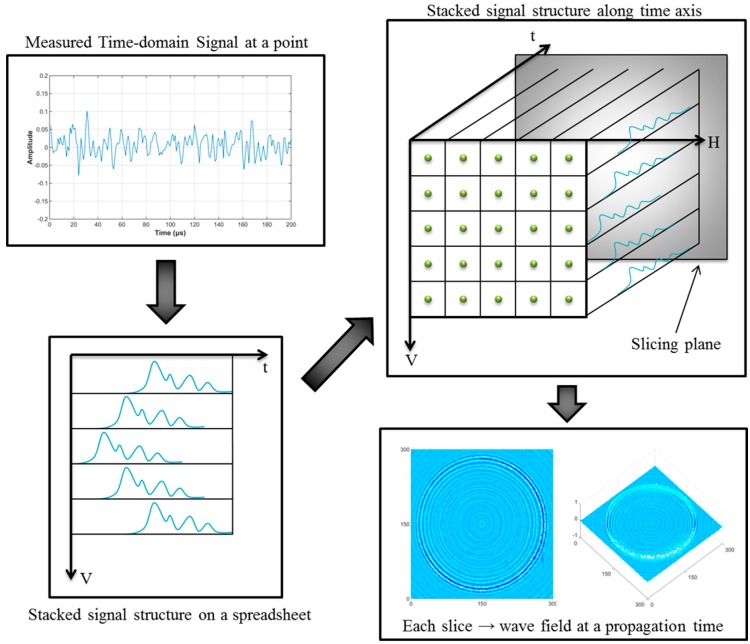
Process of the UWPI system.

During detection, the laser beams scan the target structure, and the ultrasonic waves are generated by a thermoelastic mechanism and are then propagated. An ultrasonic transducer installed on the front or back side of the structure measures the responses of the ultrasonic wave. In this study, the ultrasonic transducer is an amplifier-integrated acoustic emission (AE) sensor made of lead zirconate titanate piezoelectric ceramics. Figure 2 shows the process flow for the UWPI system. First, the signals of a wave reflected along the laser beam path are measured, and the time-domain signal is obtained at each laser impinging point. A band-pass filter was used in this step to filter the signal and improve the SNR, and filtered data for each laser beam impinging point on the vertical axis are stacked as signal groups in a vertical structure on a spreadsheet. Then, the stacked vertical data are stacked again serially on the horizontal axis of the spreadsheet. Thus, the spreadsheets can be integrated into a horizontal plane that can be considered as data for the horizontal scan. Finally, this horizontal plane can be transformed into a 3-D ultrasonic wave propagation image with three axes: the vertical scan, the horizontal scan, and the time frame [37]. This method can be used for spreadsheet data to represent snapshots depicting the time intervals of the detection process. 3-D imaging of the structural damage analysis can then be generated by playing those snapshots in quick succession [33].

## 3. Description of the Experimental Specimens and Experimental Setup

**Figure 3:**
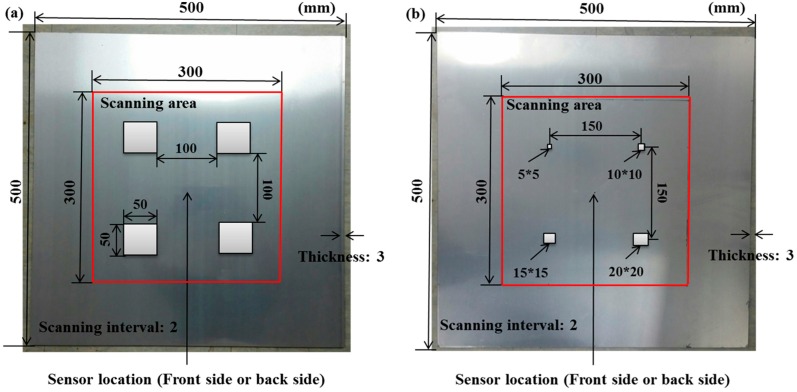
Sensor locations and scanning areas of the aluminum plate for laser scanning targets (all distances in millimeters): (**a**) intact Specimen 1; (**b**) intact Specimen 2.

As shown in Figure 3, the selected specimens were two 6061-T6 aluminum plates with a size of 500 mm × 500 mm and thickness of 3 mm. The scanned area was 300 mm × 300 mm at the central part of each specimen. In that area, the system can generate a 151 × 151 point grid (total = 22,801 points) with a 2-mm interval between points. That is, the spatial sampling rate is 2 mm. In this experiment, the scanning process was completed in 20 min. After scanning an intact specimen, corrosion damage was created on both plates using concentrated hydrochloric acid. Figure 3a shows the laser scanning area for Specimen 1. All corroded areas were the same size of 50 mm × 50 mm, but they had different depths of 0.5, 1.0, 1.5 and 2.0 mm. Figure 3b shows the laser scanning area for Specimen 2. These had the same corrosion depth but different dimensions of 5 mm × 5 mm, 10 mm × 10 mm, 15 mm × 15 mm and 20 mm × 20 mm.

The specimens were corroded by applying concentrated hydrochloric acid every day. When the concentrated hydrochloric acid was no longer applied, the damage area ceased increasing as soon as the reaction of the aluminum plate had finished. To ensure safety, the specimens were cleaned after each reaction had finished. The damage depths were measured and recorded upon completion of each corrosion reaction, and the specimens were scanned after each corrosion reaction. Since the thickness of the specimens was small, the authors scanned the back side to compare the results to those of the front side. In fact, damage is not visible on the back side, so it is more important to be able to detect damage on the back side than on the front side.

**Figure 4:**
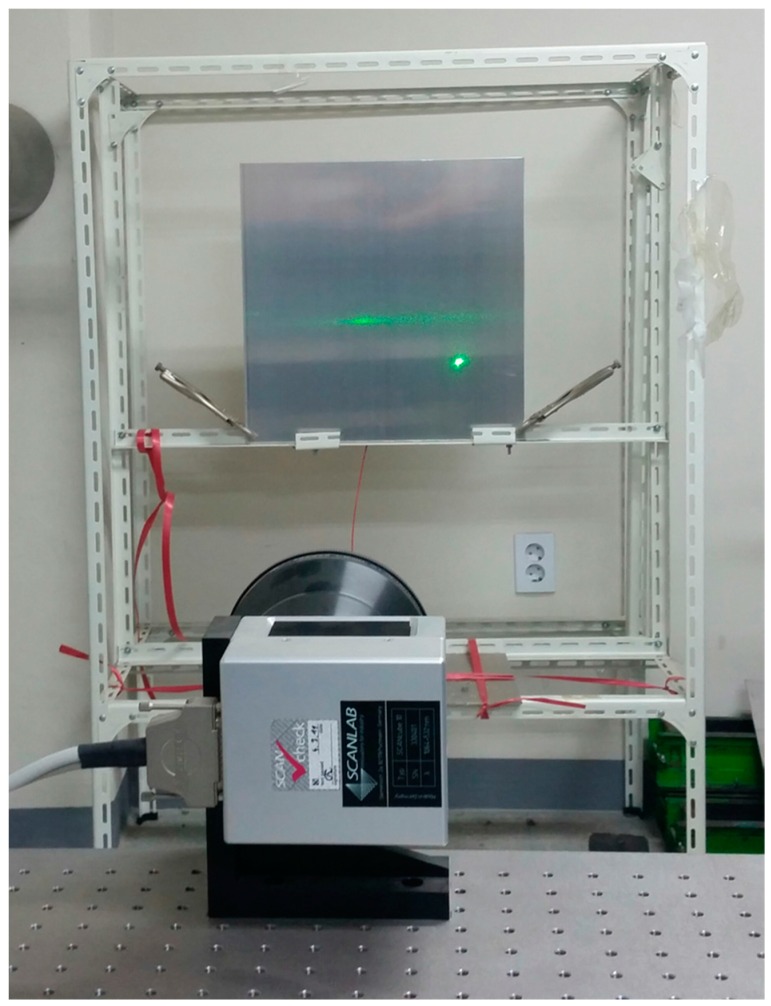
Location of fixed specimen during scanning.

The specimen was fixed on a metal support, as shown in Figure 4, tightly clamping the bottom part of the specimen with two clamps on the metal frame. An AE sensor was fixed in a central position on the back side of the measured surface to measure the multiple wave signals. The broadband AE sensor has lower and higher cut-off frequencies of 100 kHz and 2 MHz, respectively. The resonant frequency of the sensor is 200 kHz ± 20%, and at the resonant frequency, the maximum sensitivity of the sensor is 120 ± 3 dB. The scanning distance between the laser mirror scanner and the specimen was 2 m. In this circumstance, the pulsed laser was operated in an open space, and the researcher must ensure the safety of the operation via access restriction, eye protection, and adequate training.

**Figure 5:**
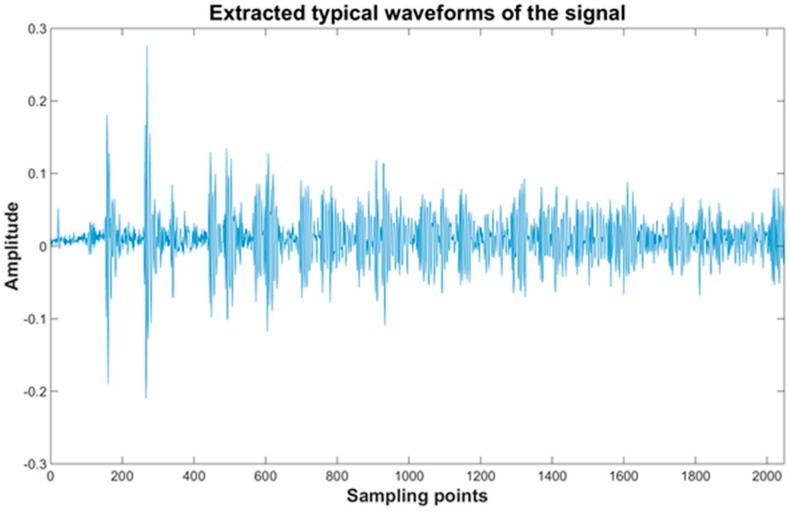
Typical waveforms of the signal measured in the intact specimen.

Figure 5 shows typical waveforms of the signal measured in the intact specimen. In general, the pulsed laser generates waves contain various frequency components, but as Figure 5 shows, the original signals already had a low noise level, so it is not necessary to apply further processing. In addition, the untreated signal can better show the test results due to the various waveforms, especially to detect some small cracks.

**Figure 6:**
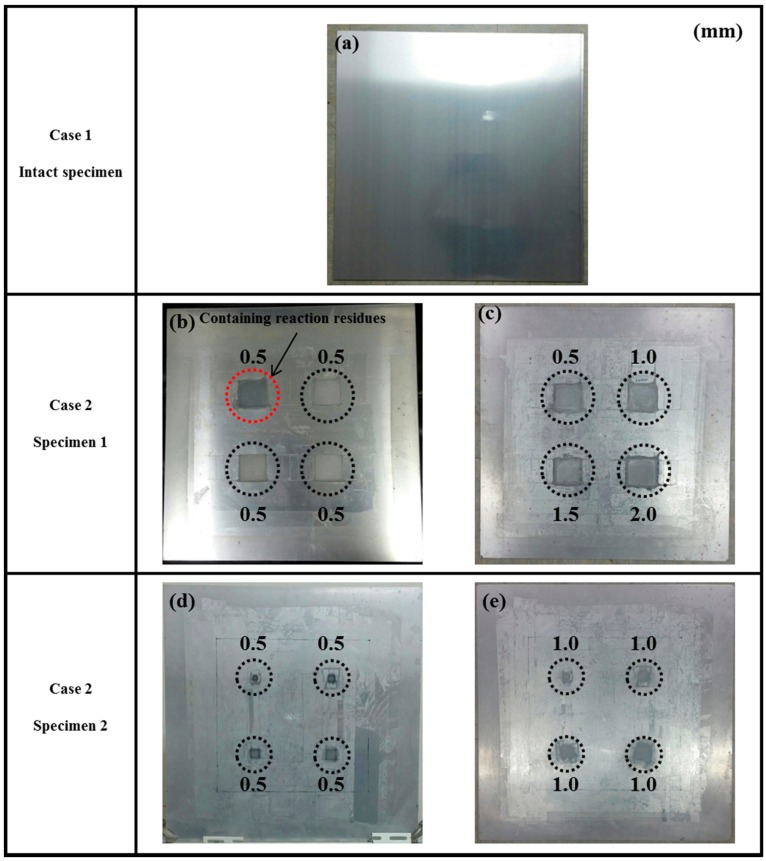
Dimensions of corroded areas: (**a**) intact specimen; (**b**) Specimen 1 (all depths = 0.5 mm); (**c**) Specimen 1 (depths = 0.5, 1.0, 1.5, and 2.0 mm); (**d**) Specimen 2 (all depths = 0.5 mm); (**e**) Specimen 2 (all depths = 1.0 mm).

Figure 6 shows the dimensions of the corroded areas. The intact specimen is shown in Figure 6a as reference for the corroded specimens. The corrosion conditions for Specimen 1 are shown in Figure 6b,c. Figure 6b shows the early stage of corrosion, where the depths of all damaged areas were 0.5 mm. It is worth noting that the specimen was not adequately cleaned after the corrosion reaction, so the top left corrosion area has some reaction residue. This was a mistake by the operator, but the experimental results of this mistake were unexpected. Therefore, in Figure 6b, the color for the top left corrosion area differs from that of the other three corrosion areas. In Figure 6c, the corrosion depths for specimen 1 were 0.5, 1.0, 1.5 and 2.0 mm. Figure 6d,e show the corrosion depths for Specimen 2, 0.5 and 1.0 mm, respectively.

## 4. Damage Detection Algorithm

The filtered UWPI data can be inversely transformed to effectively obtain the damage visualization image. The figures can be used to describe this process according to the RMS distribution of a reflected signal. The equation is given in [19]:

(1) $$w_{RMS}(x,y)={\left[\frac{1}{T}\sum_{i=1}^{T}{\left(w(x,y,t)\right)}^{2}\right]}^{\frac{1}{2}}$$

where *T* denotes the total simulation time, and *w*(*x*, *y*, *t*) are the signals of the reflected waves.

In addition, the energy distributions of standing waves are not uniform, so the color distribution in the RMS snapshots will be not uniform. To address that problem, the authors used the logarithmic values of the RMS function to ease the uniform distribution of the standing wave energy.

**Figure 7:**
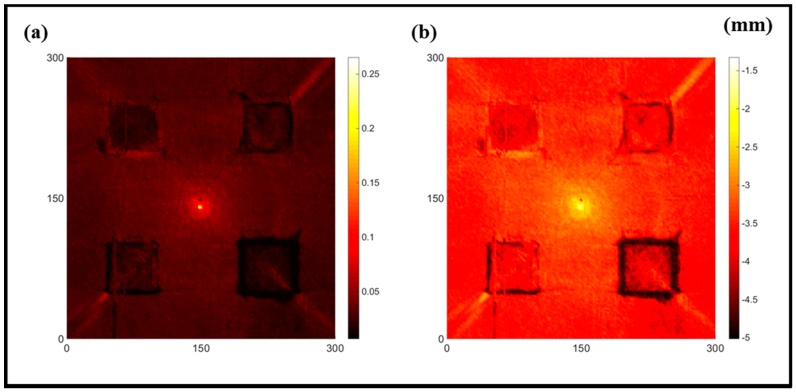
The RMS values distribution of Specimen 1 with damage depths of 0.5, 1.0, 1.5 and 2.0 mm at 400 μs (**a**) *w_RMS_*(*x*, *y*) function; (**b**) log(*w_RMS_*(*x*, *y*)) function.

Figure 7 shows the *w_RMS_*(*x*, *y*) and log(*w_RMS_*(*x*, *y*)) function snapshots of Figure 6c at 400 μs. The result in Figure 6a shows that the image was too dark, although the damaged areas were successfully detected. On the other hand, the RMS snapshots were clearly improved by using the logarithmic values of the *w_RMS_*(*x*, *y*) function, as shown in Figure 7b. Although the visual effect in Figure 7b improved significantly, the color distribution is still not sufficiently uniform. For example, more wave packet signals accumulated in the middle part of the scanning area (the sensor position) at the early stage, so the color was lighter (gold). This also signifies that in the color axis, the color becomes lighter as the RMS value increases. On the other hand, the figures also show a significant level of corrosion, and the greater depth is shown as a deeper color (a smaller value of RMS) in the RMS snapshots.

Since the accumulations of standing wave energy in the vicinity of the sensor are more frequent, larger values are produced near the location of the sensor in the RMS function. Therefore, the farther the damage is from the sensor, the harder it is to detect. However, the RMS value of the entire area can be equalized during the subsequent time period by multiplying it with the following weighting parameter:

(2) $$w_{RMS}(x,y)\_W^{k}={\left[\frac{1}{T}\sum_{i=1}^{T}{\left(w(x,y,t)\right)}^{2}\cdot t^{k}\right]}^{\frac{1}{2}}$$

where *k* is the weighting parameter and *w_RMS_*(*x*, *y*)_*W^k^* is the proposed weighted RMS function. Therefore, the RMS snapshots can be improved by using the logarithmic values of the *w_RMS_*(*x*, *y*)_*W^k^* function.

**Figure 8:**
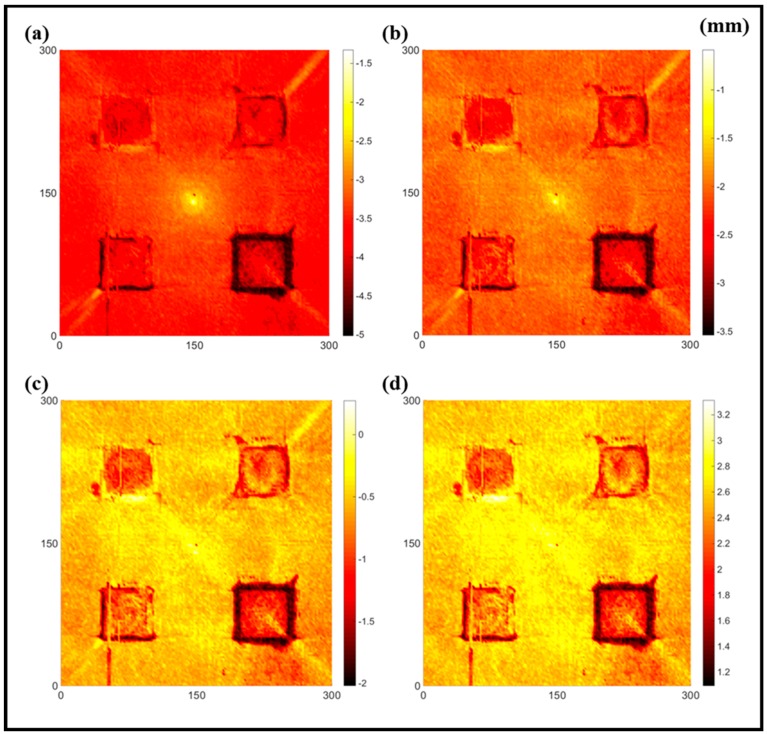
The RMS values distribution of log(*w_RMS_*(*x*, *y*)_*W^k^*) function for Specimen 1 with the depths of 0.5, 1.0, 1.5, and 2.0 mm at 400 μs: (**a**) *k* = 0; (**b**) *k* = 0.5; (**c**) *k* = 1; (**d**) *k* = 2.

Figure 8 shows the log(*w_RMS_*(*x*, *y*)_*W^k^*) function for Specimen 1 (the specimen condition in Figure 6c) when *k* = 0, 0.5, 1 and 2. The value that produces the best visual effect can easily be found through observation, which is *k* = 2. Therefore, the value of the weighting parameter is 2 in this study.

## 5. Discussion of the Experimental Results

### 5.1. Case 1: Intact Specimen

In Case 1, the intact specimen was scanned to provide a reference point for comparison with the damaged conditions. The authors used the UWPI and RMS snapshots at 60, 220, and 400 μs for this discussion because at those moments, the wave propagation phenomenon can be clearly observed. Table 1 shows the UWPI and RMS snapshots of the intact specimen at 60, 220, and 400 μs. The results show that the wave packet propagated smoothly and dispersedly from the central location, with a radial form in the circumferential boundary condition. In addition, the signal waves gradually weakened due to damping. The RMS snapshots showed a structural condition with no damage, and as the RMS value increases over time, the color of the RMS snapshots changes following the value of the color axis.

### 5.2. Case 2: Specimen 1, with All Corroded Areas the Same Size (50 mm × 50 mm)

The results detected for the two sides for Figure 6b are shown in Table 2 and Table 3. In Table 2, the circles show that the propagation of the wave packet was slower there than in the other areas, and the degrees of interference were similar. This phenomenon shows that the propagation of the wave packet was interrupted when the waves passed through the corroded areas. At 220 μs, the same phenomenon was detected. In addition, at 400 μs the result shows very confusing wave propagation, and the result is not as clear as that in Table 1.

It is worth noting that abnormal fluctuations in wave propagation were found in the marked rectangle areas, wherein the wave motion in the top left marked area was more active than in the other three areas. For the RMS snapshots, the result shows that the four corroded areas were detected at 60 μs. Although the depths of the four corroded areas were the same, the color of the top left corroded area differed from that of the other three areas. The RMS values of the other three corrosion areas were similar. The results at 220 and 400 μs showed that damaged areas were clearly observed, with increased stacking of the standing wave energy over time, even though the depth was only 0.5 mm. On the other hand, the reaction residue remaining in the top left corroded area caused its test result to differ from those of the other damaged areas, even though the depths were the same. Also, the energy of the laser increased with the temperature of the pulsed laser system, so the RMS values for the left area were larger than those for the right area. For the back side, it is worth noting that the detection takes place on the back-side surface, so the figures need to be observed in reverse. In Table 3, the results were similar to those shown in Table 2. The same phenomena were visible in the same marked positions. However, the results differ slightly because the damaged area was on the opposite side to the detection surface. Therefore, the observed phenomena were not as clear as in Table 2. The RMS snapshots from the back side show that the damaged areas could not be observed in the results at 60 μs and only became recognizable at 400 μs. Those results show that the proposed system successfully detected the corroded areas on the front and back sides, even though the depth was only 0.5 mm.

Table 4 and Table 5 show the results for Figure 6c. Table 4 shows the UWPI and RMS snapshots from the front side. At 60 μs, the circles show where the wave propagation was obviously interrupted. The influence of the interruption at the top left area (depth = 0.5 mm) was the smallest, and that at the top right area (depth = 1.0 mm) was clearly more significant. Furthermore, the reflected wave became a source of new waves when the propagating waves encountered the corroded areas. Therefore, the wave reflection phenomenon occurred at the bottom left (depth = 1.5 mm) and bottom right (depth = 2.0 mm) areas. The greater depth indicates that the reflected wave occurred at the bottom right area later than at the bottom left area. Therefore, this phenomenon can be used for damage detection. In addition, the same phenomenon occurred at 220 μs, and the wave motion on the corroded area became blurred. At 400 μs, further blurred wave motions occurred in the corroded areas. In this figure, the wave propagation in the bottom right corroded area (depth = 2.0 mm) was the most disorganized, and the results showed that the wave propagation was more severely influenced by deeper damage. The RMS results clearly showed corroded areas, even at 60 μs.

For the back side, the result in Table 5 was similar to that shown in Table 4. The wave propagation was more severely influenced by the corroded area with a greater depth, and the corroded areas in the bottom left (depth = 2.0 mm) and bottom right (depth = 1.5 mm) were clearly observed in 60 μs. At 220 and 400 μs, the color ranges from light to dark for the corroded areas in the following order: bottom left, bottom right, top left, and top right. This order follows the depth of corrosion from deep to shallow, so damage detection on the back side shows a lighter color (a larger value of RMS) as the damage becomes more severe while damage detection on the front side shows a deeper color (a smaller value of RMS) as the damage becomes more severe. The results thus show that the system successfully detected the corroded areas on both the front and back sides, and it distinguished damage at different depths.

### 5.3. Case 3: Specimen 2 with Corrosion Dimensions of 5 mm × 5 mm, 10 mm × 10 mm, 15 mm × 15 mm, and 20 mm × 20 mm

The results from the specimen shown in Figure 6d are shown in Table 6 and Table 7. The depth of the corrosion was 0.5 mm in all areas. In Table 6, no interference phenomenon was detected in the UWPI snapshots for the front side of the specimen. However, the RMS snapshots successfully display the corroded areas, even at 60 μs. For the back side, the authors also detected no interference phenomenon in the UWPI snapshots, as shown in Table 7. According to the result shown in Table 3, the system can detect damage at a depth of 0.5 mm. The RMS result at 60 μs showed four shallow traces in the wave propagation paths after passage through the corroded areas. This phenomenon thus demonstrates the existence of damage in those areas, but the dimensions of the damage cannot be measured. Unfortunately, although the system successfully observed corroded areas of the same depth in Table 3, it did not detect corroded areas clearly in Table 7, possibly because the corroded areas were too small.

The results for the specimen shown in Figure 6e are shown in Table 8 and Table 9, and the depth of the corrosion was 1.0 mm in all areas. Table 8 shows the results from the front side. At 60 and 220 μs, the marked areas indicate where the interference phenomenon occurred, and the degree of interference was similar for both cases. The RMS snapshots clearly show the corroded areas, with similar RMS values. The result for 60 μs showed that, when the waves pass through the corroded areas, the color of the RMS snapshots at the propagated area of those waves becomes lighter, indicating an increase in RMS values in those areas. For the back side, the UWPI results shown in Table 9 were very similar to those shown in Table 8. These cases thus confirm that the results on the back side were very similar to the results on front side. However, since the damaged areas were located on the front side, the image for the front side was clearer than that for the back side. Fortunately, damaged areas with a 1-mm depth were successfully detected from the back side, as shown in the RMS snapshots of Table 9. Also, the RMS values for the corroded areas were similar.

## 6. Conclusions

In this study, numerical and experimental measurements showed that the proposed method can be successfully employed to detect and locate corrosion. The proposed method allows for high-precision measurements of the Lamb wave propagation phenomenon. The RMS method is very sensitive for corrosion detection in aluminum plate and can pinpoint multiple corroded areas, even when the depth was 0.5 mm at both sides of the specimen. At the same time, it is important that the RMS method is much less sensitive to noise. Furthermore, the logarithmic values of the RMS-based functions can be used to improve the corrosion detection quality. A weighting parameter can be used to compensate for the attenuation caused by the geometrical spreading and dispersion of the waves, and this study obtained the best results using a weighting factor of *k* = 2 and the logarithmic value of the RMS-based function.

If the damage is deep enough (i.e., more than 1 mm), the reflected wave becomes a source of new waves in the UWPI snapshots when the propagating waves encounter the corroded area, whether the damage was on the front or on the back side. To detect damage on the front side, the color becomes deeper as the damage becomes more severe because the RMS value of the corroded areas decreases. However, to detect damage on the back side, the color becomes lighter as the corrosion becomes more severe because the RMS value of the corroded areas increases. Therefore, the depth of the corrosion can be distinguished. Since all damaged areas were located on the front sides of the specimens, the results for the front side were clearer than those for the back side. When the corrosion depth for Specimens 1 and 2 was 0.5 mm, the corroded areas could not be clearly detected from the back side of Specimen 2, possibly because the damaged areas were too small. It is worth noting that in Table 2, even though the depths of the four corroded areas was the same, the corrosion which has the reaction residue remaining may have the different result, but it can also be obviously detected.

This method can be applied in SHM systems with potential for high detection rates and greatly reduced operator effort and setup time. Further studies will improve the accuracy of the damage level classification as well as the visual effects for damage detection. In addition, the study will be extended for more complex structures to further verify the usability of this technique.

## Figures and Tables

**Table 1:** UWPI and RMS snapshots of the intact specimen at 60 μs, 220 μs and 400 μs (the unit of *x* and *y* axis: mm).

Time	60 μs	220 μs	400 μs
UWPI snapshots	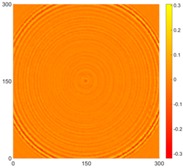	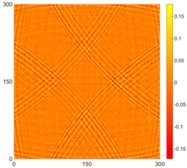	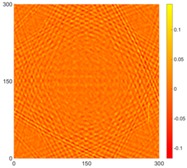
RMS snapshots	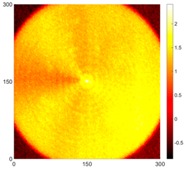	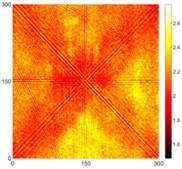	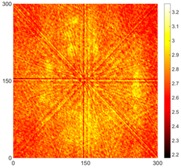

**Table 2:** UWPI and RMS snapshots of Specimen 1 front side at 60 μs, 220 μs and 400 μs (the unit of *x* and *y* axis: mm).

Time	60 μs	220 μs	400 μs
UWPI snapshots	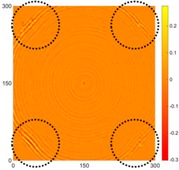	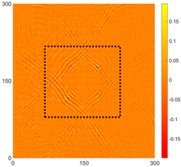	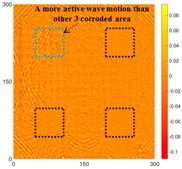
RMS snapshots	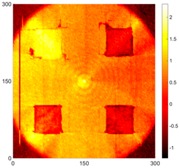	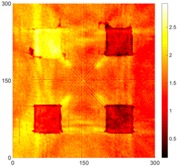	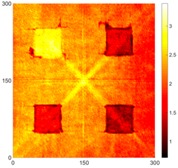

**Table 3:** UWPI and RMS snapshots of the back side of Specimen 1 at 60 μs, 220 μs and 400 μs (the unit of *x* and *y* axis: mm).

Time	60 μs	220 μs	400 μs
UWPI snapshots	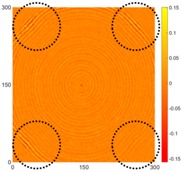	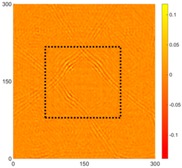	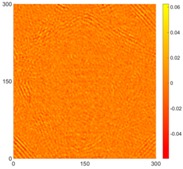
RMS snapshots	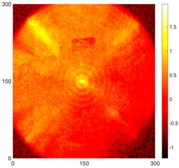	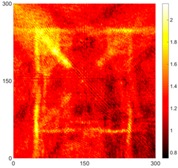	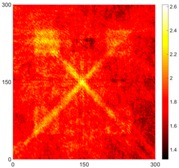

**Table 4:** UWPI and RMS snapshots of the front side of Specimen 1 at 60 μs, 220 μs and 400 μs (the unit of *x* and *y* axis: mm).

Time	60 μs	220 μs	400 μs
UWPI snapshots	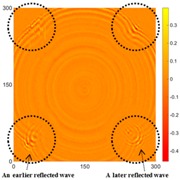	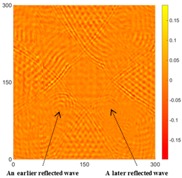	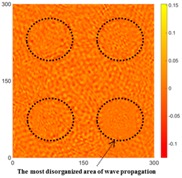
RMS snapshots	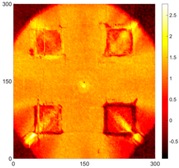	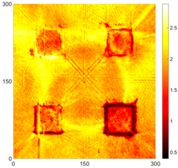	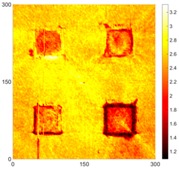

**Table 5:** UWPI and RMS snapshots of the Specimen 1 back side at 60 μs, 220 μs and 400 μs (the unit of *x* and *y* axis: mm).

Time	60 μs	220 μs	400 μs
UWPI snapshots	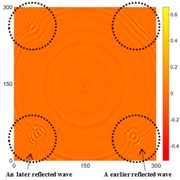	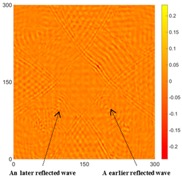	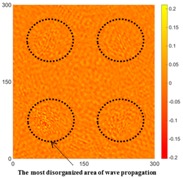
RMS snapshots	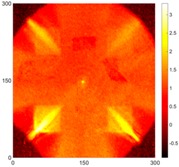	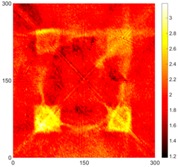	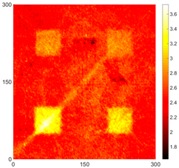

**Table 6:** UWPI and RMS snapshots of the front side of Specimen 2 at 60 μs, 220 μs, and 400 μs (the unit of *x* and *y* axis: mm).

Time	60 μs	220 μs	400 μs
UWPI snapshots	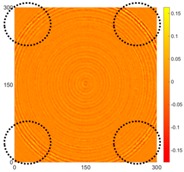	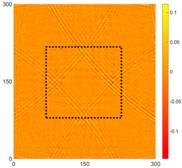	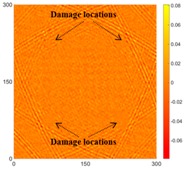
RMS snapshots	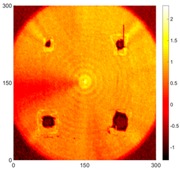	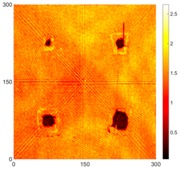	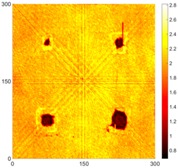

**Table 7:** UWPI and RMS snapshots of the back side of Specimen 2 at 60 μs, 220 μs and 400 μs (the unit of *x* and *y* axis: mm).

Time	60 μs	220 μs	400 μs
UWPI snapshots	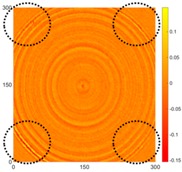	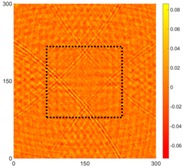	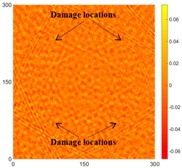
RMS snapshots	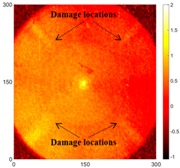	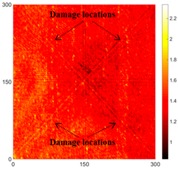	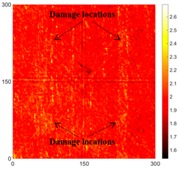

**Table 8:** UWPI and RMS snapshots of the front side of Specimen 2 at 60 μs, 220 μs and 400 μs (the unit of *x* and *y* axis: mm).

Time	60 μs	220 μs	400 μs
UWPI snapshots	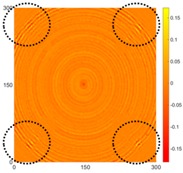	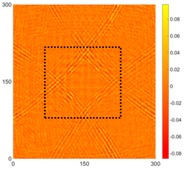	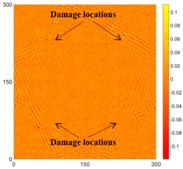
RMS snapshots	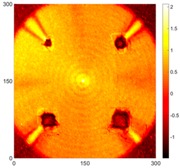	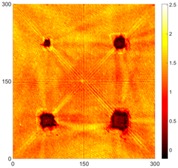	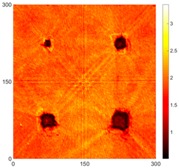

**Table 9:** UWPI and RMS snapshots of the back side of Specimen 2 at 60 μs, 220 μs and 400 μs (the unit of *x* and *y* axis: mm).

Time	60 μs	220 μs	400 μs
UWPI snapshots	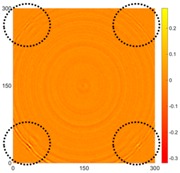	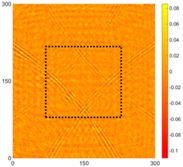	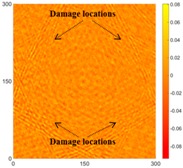
RMS snapshots	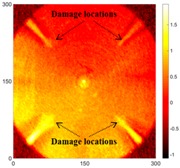	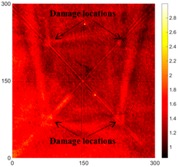	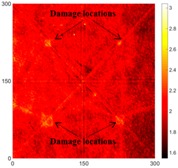

## Nomenclature

NDE	Nondestructive evaluation SHM: Structural health monitoring
PZT sensor	Piezoelectric sensor LDV: Laser Doppler vibrometer
SNR	Signal-to-noise ratio ACT: Air-coupled transducer
UWPI	Ultrasonic wave propagation image RMS: Root mean square
AE sensor	Acoustic emission sensor *w*(*x*, *y*, *t*): Signals of reflected waves
*w_RMS_*(*x*, *y*)	RMS function k: Weighting parameter
*w_RMS_*(*x*, *y*)_*W^k^*	Weighted RMS function
log(*w_RMS_*(*x*, *y*)_*W^k^*	Logarithmic values of the weighted RMS function
